# Tunneled antibiotic-impregnated vs. bolt-connected, non-coated external ventricular drainage: a comparison of complications

**DOI:** 10.3389/fneur.2023.1202954

**Published:** 2023-08-11

**Authors:** Celia Ortega-Angulo, Ana Royuela, Teresa Kalantari, Gregorio Rodríguez-Boto, Raquel Gutierrez-Gonzalez

**Affiliations:** ^1^Department of Neurosurgery, Hospital Central de la Defensa Gómez Ulla, Madrid, Spain; ^2^Biostatistics Unit, Puerta de Hierro University Hospital, Biomedical Research Institute Instituto De Investigación Sanitaria Puerta de Hierro - Segovia de Arana, Consorcio de Investigación Biomèdica en Red de Epidemiología y Salud Pública, Madrid, Spain; ^3^Department of Neurosurgery, Puerta de Hierro University Hospital, Majadahonda, Madrid, Spain; ^4^Department of Surgery, Faculty of Medicine, Instituto De Investigación Sanitaria Puerta de Hierro - Segovia de Arana, Autonomous University of Madrid, Madrid, Spain

**Keywords:** drainage, external ventricular drain, post-operative complications, ventriculostomy, anti-bacterial agents

## Abstract

**Background:**

External ventricular drainage (EVD) is a common emergency neurosurgical procedure, but it is not free of adverse events. The aim of this study is to compare the complication rate of two frequently used EVD types, namely, tunneled antibiotic-impregnated catheters (Bactiseal^©^) and bolt-connected non-coated devices (Camino^©^).

**Methods:**

All EVDs placed between 1 March 2015 and 31 December 2017 were registered. Procedures performed with any catheter different from Bactiseal^©^ or Camino^©^ EVD with incomplete follow-up and those EVDs placed due to infectious disease were excluded. Demographic and clinical variables, as well as the overall complication rate (infection, hemorrhage, obstruction, malposition of the catheter, and involuntary pull-out of the device) and the need for replacement of the EVD, were collected.

**Results:**

A total of 77 EVDs were finally considered for analysis (40 Bactiseal^®^ and 37 Camino^®^). There was a statistically significant difference in diagnosis and also in the location of the procedure, as more bolt-connected EVD was placed outside the operating room (97.3 vs. 23.5%, *p* < 0.001) due to emergent pathologies such as vascular diseases and spontaneous hemorrhages. In the univariate analysis, a statistically significantly higher rate of catheter involuntary pull-out (29.7 vs. 7.5%, *p* = 0.012) and the need for EVD replacement (32.4 vs. 12.5%, *p* = 0.035) was found in the Camino cohort. However, those differences could not be confirmed with multivariable analysis, which showed no association between the type of catheter and any of the studied complications. Ventriculostomy duration was identified as a risk factor for infection (OR 1.09, 95% CI 1.02–1.18).

**Conclusion:**

No significant differences were observed regarding infection, hemorrhage, obstruction, malposition, involuntary catheter pull-out, and the need for EVD replacement when comparing non-impregnated bolt-connected EVDs (Camino^®^) with tunneled antibiotic-impregnated catheters (Bactiseal^®^). The duration of EVD was associated with an increased risk of infection.

## Background

External ventricular drainage (EVD) is one of the most common procedures in neurosurgery. A catheter is placed in the ventricular system to monitor intracranial pressure (ICP) and drain cerebrospinal fluid (CSF) when needed. It is usually a lifesaving procedure in an emergency, but the simple technique is not complications-free. Ventriculostomy-related infection (VRI) is the most frequently addressed complication in the literature. The importance lies in the patient's outcome, the economic impact, and the hospital stay derived (1–5). The frequency rate varies widely between 0 and 45% in different studies (1–3, 6–8, 11, 13–15). Retrograde colonization of the catheter through the solution of continuity in the skin wound is considered the main mechanism of infection (6). Many factors have been associated with VRI, and many strategies have been proposed to avoid it. The most frequent ones have to do with the implementation of standardized bundle approaches (7, 8), the use of prophylactic antibiotics (9), and the replacement of the device after some days of use (2, 4, 7).

The development of antibiotic-impregnated catheters was a paradigm shift in the rates of overall EVD infection. Many studies have proven a lower risk of infection and colonization and delayed onset of VRI with this kind of device (10–12). After that, a new system to fix the EVD to the head, with a bolt attached to the skull, was developed to avoid contact between the catheter and the skin. Many studies have shown the advantages of this bolt system, with a lower infection rate, reduced risk of EVD accidental pull-out, and a higher rate of optimal position of the catheter in the lateral ventricle (13–15). Moreover, this kind of EVD is easier to place in the emergency setting outside the operating room (OR) [e.g., in the intensive care unit (ICU)], which could be helpful for critical patients who cannot be transferred (13). The space in which the procedure takes place has also been studied, but it does not significantly affect the risk of infection (16).

Both catheters (antibiotic-impregnated and bolt-connected) focus on catheter colonization to prevent infection. However, the mechanism of action is different. To the best of our knowledge, no study has specifically compared these two methods of preventing VRI. However, some have demonstrated the significant reduction in infection that antibiotic impregnation achieves when faced with plain catheters (10, 12). Others have found that infection tends to be more common in tunneled EVDs than bolt-connected ones but without statistically significant differences (14, 17–19). Only a recent meta-analysis showed that VRI was significantly less frequent in catheters anchored with the bolt system (13).

Other complications have also been assessed. Ventriculostomy-related hemorrhage has been reported to affect 1–41% of the procedures (20–22). This rate has increased since imaging tests after EVD placement has become routine. Notably, most of the hemorrhages are minimal and, therefore, only a reduced percentage present with clinical symptoms (20, 21). However, even minor punctate bleeding can become a seizure focus (22). Obstruction of the EVD is another event that can lead to re-intervention to replace the device (23). The type of catheter and fixation system can also affect the obstruction rate, and more cases of tube occlusion have been reported in tunneled EVDs (14).

The malposition of the device may also involve revision surgery or an increase in hemorrhage risk (20). Some studies have shown the benefit of the bolt system as it allows a minor variance in the insertion direction of the catheter and gives a fixed length of the intracranial segment of the tube (13, 15, 24). On the other hand, another cause of re-intervention could be accidental pull-out of the catheter, which occurs because of the manipulation of the system, movements of the patient, or when the patient is clinically agitated. Most of these events are related to the kind of fixation to the head; thus, bolt-connected catheters could be safer in this context (13–15).

This study aims to compare the complication rate depending on the type of EVD used: one antibiotic-impregnated catheter tunneled through the subcutaneous tissue (Bactiseal^®^ Codman, Johnson & Johnson, Raynham, MA, United States) and one plain catheter anchored to the skull with a bolt system (Camino^®^ Integra LifeSciences; Princeton, NJ, United States), since no study has compared two of the most used catheters in clinical practice up to date. Infection, hemorrhage, malposition, obstruction, and pull-out rates will be independently compared to determine whether one of the catheters is superior to the other. The previous literature mainly compares bolt-connected catheters to tunneled ones (plain tubes or a mixture of plain, antibiotic-impregnated, and even silver-coated tubes) or contrasts antimicrobial coatings vs. non-antimicrobial devices. This is the first study that compares two homogeneous cohorts of the types of EVDs mentioned above.

## Materials and methods

An observational study of cohorts was designed. The study was approved by the local Ethics Committee and was conducted in accordance with the 1964 Helsinki Declaration and its later amendments or comparable ethical standards. Informed consent was obtained before any invasive procedure.

### Patients' selection

**Figure 1:**
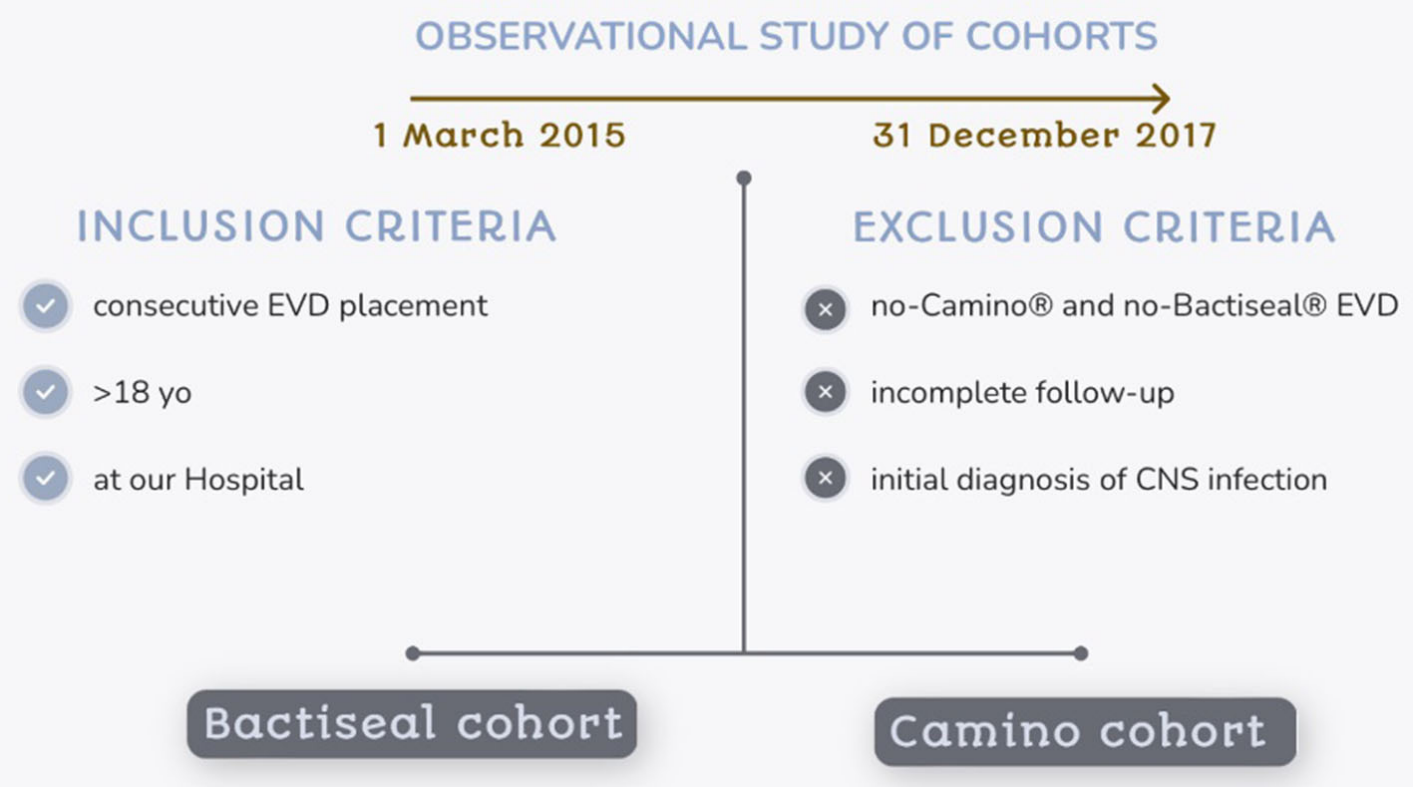
Design of the study: patients' selection.

Data were retrospectively collected. All consecutive procedures of EVD placement performed in patients aged 18 or more and performed at a tertiary hospital between 1 March 2015 and 31 December 2017 were included. The “Bactiseal cohort” comprised all procedures with antibiotic-impregnated catheters [rifampicin (0.054%) and clindamycin (0.15%)] placed and anchored by subcutaneous tunnelization. Procedures with non-impregnated catheters fixed with a bolt to the skull were classified as the “Camino cohort”. Exclusion criteria included all procedures with any other kind of catheter, incomplete follow-up, and those EVDs placed in the context of an infectious central nervous system (CNS) disease since this situation could bias the results related to the infection of the EVD device (Figure 1).

### Surgical technique and perioperative care

Even though EVD placement is not a standardized procedure in our center, all surgeons follow similar steps. The procedure is always performed in sterile conditions with specific sterilized instruments after disinfecting the skin with an iodine solution and shaving a square of hair in the area of interest. No prophylactic antibiotics are given routinely. Some patients receive antibiotics when the EVD is inserted in the OR, usually a single dose of cefazolin, or are on cefotaxime treatment as post-intubation prophylaxis. Most of the procedures are performed under general anesthesia and orotracheal intubation, as most patients are in critical situations.

**Figure 2:**
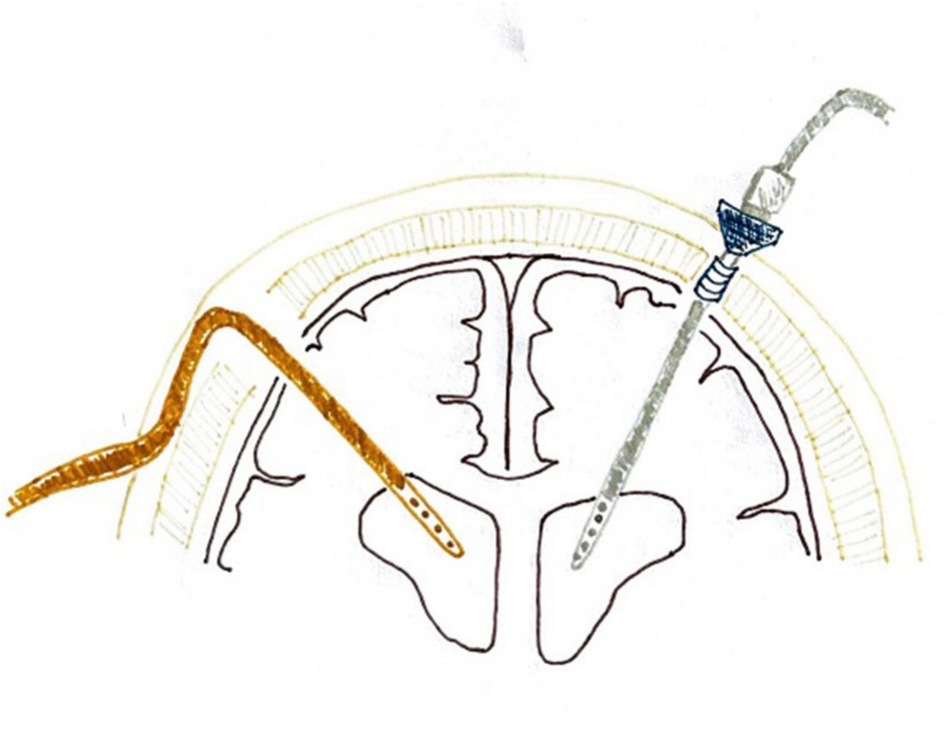
Configuration of the Camino^®^ and Bactiseal^®^ ventriculostomies. The orange catheter on the left represents a Bactiseal^®^ ventriculostomy, tunneled under the skin after exiting the cranial vault, and the gray one on the right represents the Camino^®^ system, sticking above the scalp level for some centimeters.

Antibiotic-impregnated catheters (Bactiseal^®^) are usually placed through a burr hole performed in the Kocher point (1 cm anterior to the coronal suture and 2–3 cm lateral to midline or in the mid-pupillary line), and non-impregnated catheters (Camino^®^) go through a twist-drill hole placed in the same cranial location. The right cranial side is usually preferred if the patient's pathology allows it. Both types of catheters are inserted freehand. Camino^®^ EVDs are then fixed perpendicularly to the skull with a bolt, connected to the collection system, and the small skin incision is sutured around the bolt to avoid CSF leakage. The outer part of the EVD sticks out 5–6 cm above the scalp level. Bactiseal^®^ EVDs are tunneled through the subcutaneous tissue and fixed to the skin with sutures so the external part does not stand out (Figure 2). The distance of tunnelization varies among patients and depends on the operating surgeon.

After the procedure, the skin is again disinfected, and the incision is covered with a sterile dressing or bandages. Most of the patients stay in the ICU. Only a few who are not critically ill are observed for a few hours in the post-anesthesia room and then transferred to a neurosurgical ward. CSF is not sampled unless there is clinical suspicion of infection or an unexplained fever. Post-operative computed tomography (CT) is usually performed, but no protocol standardizes the time after surgery to perform it; it depends on the patient's clinical situation, the risk of complications, and the underlying pathology. EVDs are not routinely replaced; a second placement only happens after complications (malposition, obstruction, infection, or pull-out of the device).

### Independent variables

Demographic and clinical variables were collected, including age, gender, and diagnosis. The place where the procedure was performed (OR or ICU) and the ventriculostomy duration were also noted. All data were obtained from patients' electronic health records.

### Dependent variables

#### Infection

The definition of infection was based on the presence of at least one of the meningitis/ventriculitis criteria established by the Centers for Disease Control and Prevention (CDC) and the National Healthcare Safety Network (NHSN) (25):

1. Positive CSF culture.
2. At least one of the following signs or symptoms without another identifiable cause: fever (>38°C), headache, nuchal rigidity, meningeal irritation signs, affection of cranial nerves or irritability, and at least one of the following:
  a. Increased white blood cell count, increased protein concentration, and/or diminished glucose concentration in the CSF analysis,
  b. Microorganisms observed in the Gram stain of a CSF sample,
  c. Positive blood cultures,
  d. An antigen test of a specific microorganism is positive in CSF, blood, or urine samples, or
  e. Diagnostic antibody titration (IgM) or a 4-fold increase in IgG titration for the pathogen independently of the results of the culture of the catheter.

The identity of the isolated microorganisms in cultures was registered in all cases of confirmed infection. Besides that, the VRI diagnosis date was recorded to calculate the number of days elapsed between the ventriculostomy and the complication.

#### Hemorrhage

Hemorrhage was defined as blood in the epidural or subdural spaces subjacent to the burr hole of the ventriculostomy or any blood along the ventricular catheter trajectory that was absent before the procedure. Bleeding size or volume was not considered, and the complication was registered regardless of clinical relevance.

#### Obstruction

EVD device obstruction was noted when it was specifically mentioned in the patient's clinical records.

#### Malposition

Post-operative imaging studies [CT or brain magnetic resonance imaging (MRI)] were used to evaluate the position of the EVD. Malposition was defined as the location of the tip of the catheter outside the ventricular system or as the unconventional trajectory of the catheter when the tip was in the intraventricular space.

#### Accidental pull-out

Even when correct fixation to the head of the patient was confirmed during the procedure (with a bolt system in the Camino cohort and with sutures after subcutaneous tunnelization in the Bactiseal cohort), some of the EVD suffered from accidental pull-out. This complication was registered whenever the catheter was removed unintentionally (by the patient or any healthcare worker). Not all patients required reinsertion of the EVD. However, accidental pull-out was considered a complication since it could have consequences and interfere with the patient's outcome.

#### Replacement of EVD

The need for EVD replacement, independently of the complication that motivated this subsequent procedure, was also noted.

### Statistical analysis

Database information was processed and analyzed using StataCorp. 2019 (Stata Statistical Software: Release 16. College Station, TX: StataCorp LLC). Numerical variables represented by the mean and standard deviation (SD) were contrasted using the Student's *t*-test. In contrast, those represented by the median (percentiles 25 and 75 as dispersion measures) were contrasted using the Mann-Whitney *U*-test. The chi-square test was used for categorical variables (absolute and relative frequencies as the description measure). All percentages were calculated per procedure. A secondary multivariable analysis was performed to identify factors influencing complications risk. Finally, a Cox regression analysis was employed to estimate the association between the catheter type and the moment the infection appeared.

Every statistical hypothesis was two-tail tested. The null hypotheses with a type I error or α error < 0.05 were rejected in all hypothesis contrasts.

## Results

**Figure 3:**
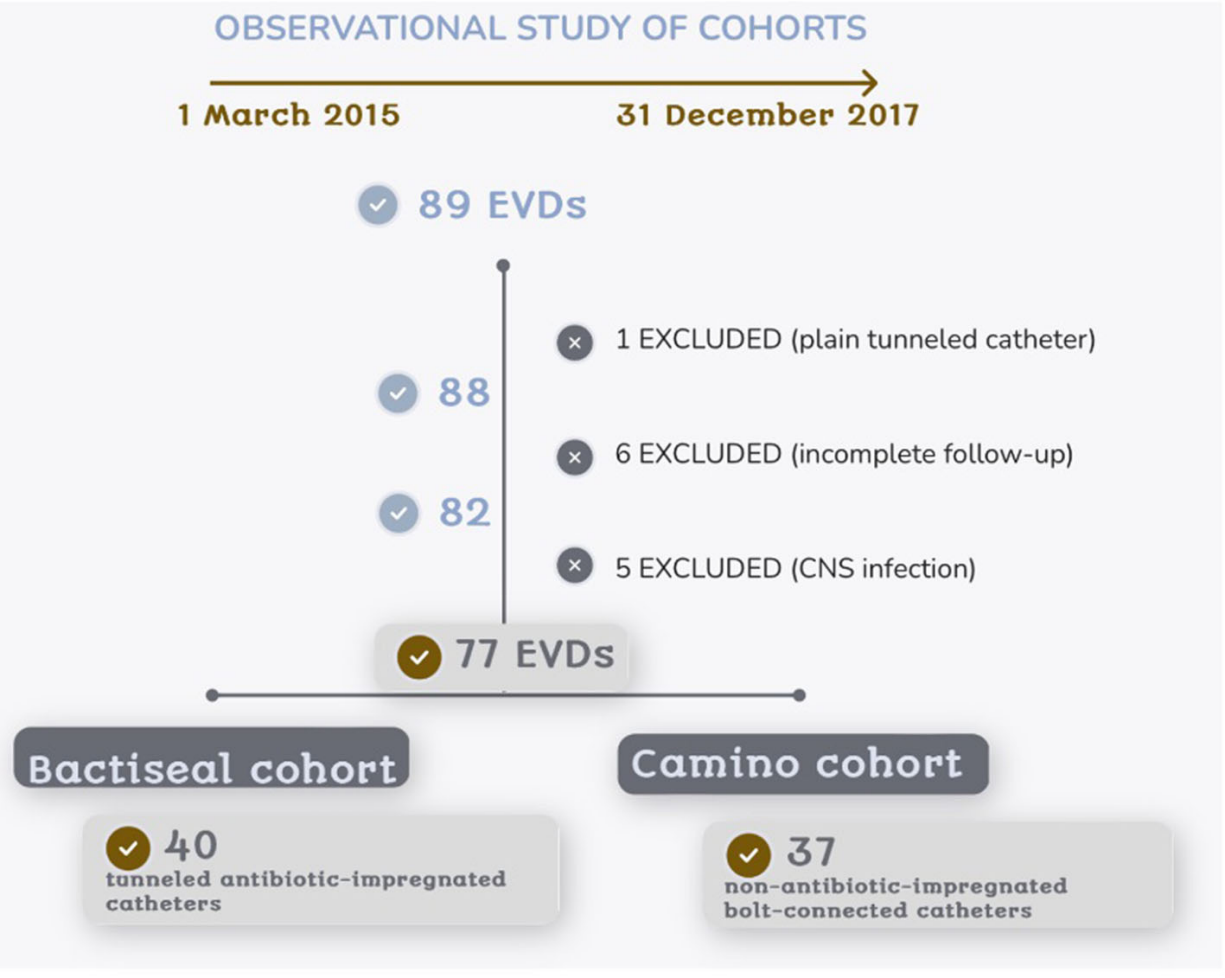
Conformation of the studied cohorts after exclusion criteria.

A total of 89 procedures were performed during the study period. Follow-up was incomplete in six cases, and another five procedures were performed due to an infectious CNS disease. Besides that, one procedure was excluded since a tunneled plain catheter (no-Bactiseal^®^, no-Camino^®^ EVD) was used, thus 77 EVDs were finally considered for analysis. Non-antibiotic-impregnated catheters connected with a bolt to the skull (Camino^®^) were used in 37 of these procedures, and 40 antibiotic-impregnated catheters (Bactiseal^®^) were used in the remaining cases (Figure 3). Both cohorts were similar in age and gender distribution (Table 1). Spontaneous intracerebral or intraventricular hemorrhage and vascular pathology were more common diagnoses in the Camino cohort (40.5 and 51.4%, respectively, vs. 15.0 and 40.5% in the Bactiseal cohort), and tumor pathology was exclusively seen in the Bactiseal cohort (32.5%). Most of the antibiotic-impregnated tunneled EVDs (Bactiseal^®^) were placed in the OR (77.5%), but only a few of the Camino^®^ EVDs (2.7%), with a statistically significant difference (*p* < 0.001). There was no significant difference in ventriculostomy duration between the two cohorts, with a median of 10.5 days in the Bactiseal group and 8 days in the Camino cohort.

**Table 1:** Characteristics of EVD procedures: comparison between the two cohorts.

**Variables**	**Bactiseal cohort (*n* = 40)**	**Camino cohort (*n* = 37)**	***p***
Age [mean (SD)]	56.4 (12.9)	56.5 (12.2)	0.976
Male patient [*n* (%)]	29 (72.5)	26 (70.3)	0.829
EVD placed in OR [n (%)]	31 (77.5)	1 (2.7)	< 0.001
Days of ventriculostomy [median (IQR)]	10.5 (4;5)	8 (3;17)	0.810
Diagnosis [*n* (%)]			0.002
Tumor pathology	13 (32.5)	0 (0)	
Vascular pathology	16 (40.0)	19 (51.4)	
Traumatic brain injury	2 (5.0)	1 (2.7)	
Spontaneous hemorrhage	6 (15.0)	15 (40.5)	
Other	3 (7.5)	2 (5.4)	

SD, standard deviation; EVD, external ventricular drain; OR, operating room; IQR, interquartile range.

Infection was diagnosed in seven of the 77 EVDs, with an overall infection rate of 9.1%. In total, nine cases of hemorrhage were identified (11.7%), 15 events of obstruction (19.5%), and seven of catheter malposition (9.1%). Accidental pull-out happened in 14 of the devices (18.2%), and EVD replacement was needed in 17 cases (22.1%) after any of the complications mentioned above. Thus, the overall complication rate was 50.6%. Accidental pull-out was more common in the Camino cohort (29.7 vs. 7.5% in the Bactiseal cohort, *p* = 0.012). Then, EVD replacement was also more frequent in the Camino cohort (32.4 vs. 12.5% in the Bactiseal cohort, *p* = 0.035). There was a trend toward higher rates of infection and hemorrhage in the Camino cohort and a higher rate of malposition in the Bactiseal cohort, in the absence of statistical significance in all cases. No differences were determined in the EVD obstruction rate. Comparisons between both cohorts are summarized in Table 2.

**Table 2:** Comparison of complication rates: univariable analysis.

**Variables**	**Bactiseal cohort (*n* = 40)**	**Cami*n*o cohort (*n* = 37)**	***p***	**Odds ratio (95% CI)**
Infection [*n* (%)]	2 (5.0)	5 (13.5)	0.194	2.97 (0.54–16.3)
Hemorrhage [*n* (%)]	2 (5.0)	7 (18.9)	0.058	4.43 (0.86–22.9)
Obstruction [*n* (%)]	7 (17.5)	8 (21.6)	0.648	1.30 (0.42–4.0)
Malposition [*n* (%)]	5 (12.5)	2 (5.4)	0.279	0.40 (0.07–2.2)
EVD pull-out [*n* (%)]	3 (7.5)	11 (29.7)	**0.012**	**5.22** (1.32–20.6)
Overall complication [*n* (%)]	17 (41.5)	22 (59.5)	0.137	1.98 (0.80–4.9)
EVD replacement [*n* (%)]	5 (12.5)	12 (32.4)	**0.035**	**3.36** (1.05–10.7)

EVD, external ventricular drain; CI, confidence interval. The bold values indicate the *p*-value < 0.05.

A multivariable analysis was performed to examine the influence of individual factors on the risk of the studied complications (Table 3). No significant difference was confirmed between the Bactiseal and Camino cohorts in any of the aforementioned adverse effects. However, an increased risk of infection was evidenced in prolonged ventriculostomies [odds ratio (OR) 1.09, 95% confidence intervals (CI) 1.02–1.18]. A strong association was evidenced between intracranial bleeding (comprising the diagnosis of spontaneous hemorrhage and vascular pathology) and the risk of obstruction, with borderline statistical significance (*p* = 0.053). Long-lasting ventriculostomies were also associated with a smaller risk of pull-out (OR 0.89, 95% CI 0.81–0.99), overall complications (OR 0.94, 95% CI 0.90–0.99), and EVD reinsertion (OR 0.85, 95% CI 0.75–0.95).

**Table 3:** Multivariable analysis.

**Complication factor**	***p-*value**	**Odds ratio (95% CI)**
**Infection**		
EVD camino	0.925	1.13 (0.09–14.08)
Age	0.373	0.96 (0.87–1.05)
Male gender	0.298	4.35 (0.27–69.27)
Diagnosis (intracranial hemorrhage)	0.994	2.83e+7 (0.00–inf)
ICU placement	0.421	3.59 (0.16–80.61)
Duration of ventriculostomy, days	**0.015**	1.09 (1.02–1.18)
**Hemorrhage**		
EVD Camino	0.576	1.98 (0.18–21.47)
Age	0.85	0.99 (0.93–1.06)
Male gender	0.464	0.53 (0.10–2.87)
Diagnosis (intracranial hemorrhage)	0.426	0.43 (0.06–3.40)
ICU placement	0.231	6.17 (0.31–120.97)
Duration of ventriculostomy, days	0.311	0.96 (0.88–1.04)
**Obstruction**		
EVD Camino	0.88	1.15 (0.18–7.42)
Age	0.554	0.98 (0.93–1.04)
Male gender	0.696	1.33 (0.32–5.53)
Diagnosis (intracranial hemorrhage)	0.053	9.14 (0.97–85.72)
ICU placement	0.522	0.53 (0.08–3.69)
Duration of ventriculostomy, days	0.492	1.02 (0.96–1.08)
**Malposition**		
EVD Camino	0.6	0.51 (0.04–2.02)
Age	0.383	0.97 (0.92–1.03)
Male gender	0.843	0.83 (0.13–5.19)
Diagnosis (intracranial hemorrhage)	0.217	0.30 (0.05–2.02)
ICU placement	0.736	1.50 (0.14–15.95)
**Pull-out**		
EVD camino	0.164	5.02 (0.52–48.62)
Age	0.326	1.03 (0.97–1.10)
Male gender	0.13	5.64 (0.60–52.92)
Diagnosis (intracranial hemorrhage)	0.748	1.43 (0.16–12.72)
ICU placement	0.93	1.12 (0.08–15.61)
Duration of ventriculostomy, days	**0.048**	0.89 (0.81–0.99)
**Overall complication**		
EVD camino	0.558	1.57 (0.35–7.17)
Age	0.32	0.98 (0.94–1.02)
Male gender	0.265	1.89 (0.62–5.78)
Diagnosis (intracranial hemorrhage)	0.271	1.98 (0.59–6.68)
ICU placement	0.877	1.13 (0.24–5.30)
Duration of ventriculostomy, days	**0.045**	0.94 (0.90–0.99)
**EVD replacement**		
EVD Camino	0.211	3.82 (0.47–31.29)
Age	0.839	0.99 (0.94–1.05)
Male gender	0.163	3.66 (0.59–22.61)
Diagnosis (intracranial hemorrhage)	0.068	9.68 (0.84–111.02)
ICU placement	0.397	0.36 (0.04–3.84)
Duration of ventriculostomy, days	**0.005**	0.85 (0.75–0.95)

The bold values indicate the *p*-value < 0.05.

Regarding infection parameters, the most common causal microorganisms were *Staphylococcus spp*., identified in four of the seven cases (57.1%). They all belonged to the Camino cohort (4/5, 80%), finding a significant difference between the two cohorts (*p* = 0.046). One of the infections in the Bactiseal cohort was caused by *Streptococcus* spp., and the other two cases (one in each cohort) had negative cultures despite complying with the other criteria of VRI. There were no infections by gram-negative microorganisms in any of the cohorts. The median number of days of ventriculostomy until the diagnosis of infection was 8.5 days in the Bactiseal cohort and 11 days in the Camino cohort (Table 4). However, there was no statistical difference between both groups (*p* = 0.564).

**Table 4:** Comparison of days of ventriculostomy until the development of infection.

	**Mean**	**Standard deviation**	**Median**	**IQR**
Bactiseal cohort	8.5	3.5	8.5	7.25–9.75
Camino cohort	13.4	10.5	11	7–16

IQR, interquartile range. *p* = 0.564.

## Discussion

External ventricular drainage placement is one of the most common procedures performed in Neurosurgery, but some aspects are still controversial in the literature. In this study, we have compared two types of catheters and their complication rates. In the two cohorts, which are very similar regarding demographic variables (age and gender of the patients), there was a difference in clinical diagnoses. Thus, tumor pathology was only seen in the Bactiseal cohort because most drains were placed while the surgeon acted over the tumor. Therefore, the procedure occurred in the OR. However, vascular pathology and spontaneous bleeding were more common in the Camino cohort, as the ventriculostomy was usually made as a sole procedure and took place in the ICU because it is generally performed in an emergency setting. A similar distribution of diagnosis has been described in other studies: Foreman et al. (26) showed a significant difference in EVD placed because of tumors, which happened more frequently in the OR; Roach et al. (17) used a higher rate of bolt-connected EVD in hemorrhagic pathology.

The overall infection rate observed in this series (9.1%) is in the lower half of the wide range (0–45%) described in the literature (1–3, 5–8, 10, 12, 14–19, 26–29). A higher percentage of infections in the Camino cohort was observed in our study. In contrast, most studies comparable to it (which compare bolt systems against tunneled plain catheters or a mixture of plain and antibiotic-impregnated tubes) have found better VRI rates in bolt systems (13, 15, 17–19). However, no statistically significant difference has been observed in any case.

Extensive evidence shows that coated catheters diminish VRI rates (8, 10, 12), so this fact could be responsible for the discordant results hereby observed. Other involved factors could be the setting where the EVD is placed; although non-significant, some studies have found a higher rate of VRI in procedures outside the OR (3, 12, 26, 27). Brand et al. (28) also reported a trend of more infections in the EVD placed through a twist drill (used for the bolt system) vs. the ones placed through a burr hole. On the other hand, intracranial hemorrhage (especially intraventricular but also subarachnoid hemorrhage) has been described as a risk factor for EVD infection (6, 17, 29), an association that was not found after multivariable analysis.

The pathogenesis of most VRI must be retrograde colonization of the catheter. Microorganisms penetrate through the distal point of insertion of the EVD and go along the tube to finally reach the column of CSF (6). Although the screw avoids contact with the subcutaneous tissue, there is still a possibility that environmental and skin bacteria could colonize the device. Both types of catheters used in this study have their own formula to combat infection: antibiotic coating in Bactiseal^®^ EVDs and avoiding contact with the skin with the bolt system in the Camino^®^ tubes. No difference in VRI was observed when comparing both catheters, even though the mechanism of action differs.

However, multivariable analysis showed an increased risk of infection in prolonged ventriculostomies (even though the duration of ventriculostomy was not significantly different between cohorts). That is a matter of controversy, as some studies have also demonstrated that ventriculostomy duration is not a risk factor for infection (29, 30). Still, others defend it as one of the most important (3–7, 9, 27). The time from ventriculostomy to VRI diagnosis is also variable in the published literature. Our data are consistent with what has been reported between 3 and 8 days in one study (30) and around 11 days in another (28). Coating the catheter with antibiotics (11) and subcutaneous tunnelization (1) have been described as factors that could delay bacterial colonization of the catheter. However, no effect was detected in this study from the moment the infection appears, depending on the type of catheter.

As previously reported, *Staphylococcus* spp. were the most frequent causal microorganisms isolated (3, 6). All infections caused by these bacteria occurred in the Camino cohort (non-impregnated catheters), observing a significant difference in favor of the Bactiseal cohort. This finding must be related to the protective effect against gram-positive bacteria that the antibiotics covering the catheter have. Likewise, all those ventriculostomies were performed in the ICU, which was also described as a risk factor for gram-positive microorganism infection (27).

The overall EVD-related hemorrhage rate was 11.7%. This complication has been described to affect 1–41% of the procedures (13, 15, 18–22, 26, 28, 29, 31). Bauer et al. (31) published a meta-analysis showing an upward hemorrhage tendency when postoperative CT was routinely performed, with an overall hemorrhage rate of 12.1% in those studies with imaging tests after the procedure, very similar to the one now reported. The EVD-related hemorrhage rate was higher in the Camino cohort, but there was no statistically significant difference after the multivariable analysis when hemorrhagic diagnosis and ICU placement were considered. There are mixed results concerning this point in the published literature. Some studies have also seen this non-significant trend (19), and others have described a significantly higher bleeding rate in tunneled catheters placed with a mechanical drill (15). Other studies have reported higher rates of EVDs placed in the ICU (22, 26) or in patients whose diagnosis was intracranial bleeding (21). However, no association was observed in this study after multivariable analysis.

The overall malposition rate (9.1%) was consistent with the literature or even lower than the rate described in most studies (13, 15–20, 24, 28, 30, 32). As with infection, the heterogeneity in definitions must be contemplated. Some studies only considered “malposition” when the EVD did not work and needed to be replaced (30); others created their grading system (24, 32, 33) or their way of defining the “optimal position” of the catheter (17, 28). In this study, there is a non-significantly higher malposition rate in the Bactiseal cohort, in accordance with that observed in tunneled EVDs (13, 15, 16, 18, 19, 24). A lower rate of malposition and the number of attempts with the bolt system have been related to the burr hole size and the variance in the insertion direction (15). Also, most bolt systems allow fixing the intracranial catheter length before implanting it (13, 24), which could reduce the mispositioning of the tip.

Malposition has been frequently associated with other complications such as infection (23), hemorrhage (20, 23, 33), obstruction (19, 33, 34), or re-intervention (7, 19, 26, 30, 33). However, in this study, the malposition rate was higher in the Bactiseal cohort, but the rest of the events (except obstruction) were less frequent. There is no apparent reason to justify these results. The lower malposition rate (compared with most published studies) may interfere with or mask the results observed.

No difference was observed in obstruction (an overall rate of 19.5%) between both cohorts. Other authors have also reported similar rates of EVD occlusion (16, 30). We did not differentiate between temporal and permanent obstructions, so the rate may be higher than that reported in other studies that only considered the cases that needed surgical revision (19, 29). Fargen et al. (34) described 42% of at least one event of temporal occlusion and 19% of permanent obstruction. They identified therapeutic anticoagulation, the non-ideal position of the catheter, and narrower tubes as risk factors. Camino^®^ EVDs have an inner diameter of 2.2 mm, and Bactiseal^®^ catheters have one of 1.4 mm, but these data do not seem to influence our results. A strong association between intracranial bleeding (comprising the diagnosis of spontaneous hemorrhage and vascular pathology) and the risk of obstruction was evidenced in the multivariable analysis, with an OR of 9.14, even though differences were marginally significant (*p*=0.053). This result could be explained since catheter occlusion is usually caused by blood clots and cellular debris (23, 35), and similar or higher rates of obstruction have been described in patients with hemorrhagic diagnoses (36).

Accidental EVD pull-out has probably been infra-reported in the literature (37), even when it can represent a risk for the patient (associated with reintervention) and may negatively affect the use of material resources (38). The overall rate of 18.2% now reported is much higher than that published in other studies (13, 14, 19, 29, 30). In our experience, the complication was more frequent in the Camino cohort, in contrast with other studies where the bolt system consistently reported better results (13, 14), achieving rates below 2% in some cases (19, 29). To the best of our knowledge, the higher rate reported is 24%, but it was found in the tunneled catheters against a solid 0% in bolt-connected EVDs (14). On the contrary, Schödel et al. (15) initially registered more dislocations in the bolt group. Still, they considered not tightening the screw enough to be the cause of these results as part of the learning curve, and their numbers decreased substantially with training. In our experience, the high rate could also be related to a similar cause in the technique (even though the problem with screw tightening was not noticed in most of the cases, and this device had been used for a long time before the study period) or be a consequence of non-standardized management and care of EVDs in the ICU. Nevertheless, we agree with previous studies that improving the technique could be a better way of diminishing involuntary pull-out of the intraventricular catheters (15, 37–39).

Reintervention, or EVD replacement, is a consequence of the mentioned complications. The overall rate in this study, 22.1%, is again higher than the ones reported in the literature, which range from 6.5 to 22% (7, 13, 14, 24, 26). However, replacement rates could be higher than described, as many studies reference a mean of 1.3 catheters per patient, approximately (23). Thus, Fargen et al. (34) described high numbers of revision surgeries with 33 catheter exchanges in 19 patients who suffered at least one episode of permanent tube obstruction. They attributed that result to more rigorous prospective data monitoring.

The need for replacement was more frequent in the Camino cohort in this study, which must be related to the higher rates of pull-out, hemorrhage, and infection in this group. However, multivariable analysis showed no statistically significant difference. The literature reports more reinterventions in tunneled catheters (13, 14, 19). Only one study by Roach et al. (17) reports non-significantly higher rates in bolt-connected EVDs (3.4% against 3% in the tunneled group).

Finally, ventriculostomy duration was significantly associated with a lower risk of pull-out, overall complications, and EVD reinsertion. The absence of adverse events can be related to a more prolonged ventriculostomy, avoiding the need for EVD replacement.

As a significant difference in complications is yet to be found between the two cohorts analyzed, the prices of both types of catheters between 2015 and 2017 were consulted. Camino^®^ EVD cost was 485€, and Bactiseal^®^ catheters were priced at 286€. Other monetary costs must be added to both procedures, like the cranial access kit used to perform bedside EVD (285€) or the cost of OR occupation (183€ every 15 min).

### Limitations of the study

The main limitation of the study is related to the small sample size. The statistical power may be higher with an increase in the number of EVDs. Another limitation is the nature of the study, which is observational and non-randomized. The retrospective design prevented analyzing some factors influencing the risk of infection (such as CSF leakage, use of antibiotics, or duration of the procedures) due to inconsistent reports in the electronic health records. Finally, we did not use standardized definitions or grading systems for all the variables analyzed. The lack of homogeneity among articles investigating EVD complications in all published literature contributes to the controversial evidence about risk factors and ways to avoid them.

Further studies with bigger sample sizes and a randomizing system should be proposed. Moreover, the difference between the two groups that we studied was not only their fixing method to the head, but also that one of them was coated with antibiotics and the other was a plain catheter. This fact makes it interesting to design a study that could compare the antibiotic-impregnated tunneled catheter with a bolt-connected EVD coated with antibiotics or antiseptics.

## Conclusion

No significant differences were observed regarding infection, hemorrhage, obstruction, malposition, involuntary catheter pull-out, and the need for EVD replacement when comparing non-impregnated bolt-connected EVDs (Camino^®^) with tunneled antibiotic-impregnated catheters (Bactiseal^®^). The duration of the ventriculostomy was associated with an increased risk of infection, but no difference was attributed to the catheter type. Further studies are needed to better characterize such complications, their causes, and risk factors.

## Data availability statement

The original contributions presented in the study are publicly available. This data can be found here: https://zenodo.org/record/8219031.

## Ethics statement

The studies involving human participants were reviewed and approved by Ethics Committee of Puerta de Hierro University Hospital. The patients/participants provided their written informed consent to participate in this study.

## Author contributions

CO-A made a substantial contribution to the conception and design of the work, the acquisition, analysis, and interpretation of data, drafted the article, and revised it. AR participated in the design of the study, performed statistical analysis, and revised the manuscript for intellectual content. TK and GR-B performed data collection and a critical review of the manuscript for intellectual content. RG-G conceived the study, participated in its design, carried out data collection, and substantively contributed to the writing and revision of the manuscript. All the authors have approved the submitted version and have agreed both to be personally accountable for the author's own contributions and to ensure that questions related to the accuracy or integrity of any part of the work, including ones in which the author was not personally involved, are appropriately investigated, resolved, and the resolution documented in the literature.
